# Exploring Optimized Spiking Neural Network Architectures for Classification Tasks on Embedded Platforms

**DOI:** 10.3390/s21093240

**Published:** 2021-05-07

**Authors:** Tehreem Syed, Vijay Kakani, Xuenan Cui, Hakil Kim

**Affiliations:** 1 Electrical and Computer Engineering, Inha University, 100 Inha-ro, Nam-gu, Incheon 22212, Korea; tehreem@inha.edu; 2 Integrated System and Engineering, School of Global Convergence Studies, Inha University, 100 Inha-ro, Nam-gu, Incheon 22212, Korea; vjkakani@inha.ac.kr; 3 Information and Communication Engineering, Inha University, 100 Inha-ro, Nam-gu, Incheon 22212, Korea; xncui@inha.ac.kr

**Keywords:** deep convolutional spiking neural networks, spiking neuron model, surrogate gradient descent, time-steps, embedded platform

## Abstract

In recent times, the usage of modern neuromorphic hardware for brain-inspired SNNs has grown exponentially. In the context of sparse input data, they are undertaking low power consumption for event-based neuromorphic hardware, specifically in the deeper layers. However, using deep ANNs for training spiking models is still considered as a tedious task. Until recently, various ANN to SNN conversion methods in the literature have been proposed to train deep SNN models. Nevertheless, these methods require hundreds to thousands of time-steps for training and still cannot attain good SNN performance. This work proposes a customized model (VGG, ResNet) architecture to train deep convolutional spiking neural networks. In this current study, the training is carried out using deep convolutional spiking neural networks with surrogate gradient descent backpropagation in a customized layer architecture similar to deep artificial neural networks. Moreover, this work also proposes fewer time-steps for training SNNs with surrogate gradient descent. During the training with surrogate gradient descent backpropagation, overfitting problems have been encountered. To overcome these problems, this work refines the SNN based dropout technique with surrogate gradient descent. The proposed customized SNN models achieve good classification results on both private and public datasets. In this work, several experiments have been carried out on an embedded platform (NVIDIA JETSON TX2 board), where the deployment of customized SNN models has been extensively conducted. Performance validations have been carried out in terms of processing time and inference accuracy between PC and embedded platforms, showing that the proposed customized models and training techniques are feasible for achieving a better performance on various datasets such as CIFAR-10, MNIST, SVHN, and private KITTI and Korean License plate dataset.

## 1. Introduction

Deep learning is utilized to perform numerous responsibilities, for instance, image recognition, detection, and speech recognition [1,2,3]. These modern developments in deep learning have provided new possibilities for designing various engineering demands and perceptions of how the biological brain works [4]. Such deep learning methods yield valuable advancements in traditional artificial neural networks by producing distinct levels of hierarchical structures and exhibiting extraordinary results that sometimes exceed human-level capability [5]. Due to such developments, it is used on large-scale computer systems, medical devices, and robots. In accordance with a large amount of deep learning data, it needs considerable energy demands on the modern servers.

Spiking neural networks (SNNs) are biologically inspired neurons, and these neurons communicate in the form of a sequence of spikes. The network consists of spiking neurons that send data in the form of a small number of spikes [6]. SNNs as neuromorphic computing has been quite common for achieving energy-efficiency in the context of standard artificial intelligence tasks [7]. SNNs are used for the classification of Motor Imagery Movements from EEG Signals [8], Multivariate Olfaction [9], Odor Data [10], Surface electromyography (sEMG) [11] and Emotions [12].The computational power of SNNs is hypothetically no less than that of artificial neural networks (ANNs) [13]. SNNs are usually considered the third generation of artificial neural networks, and they have some differences. One difference is that the spiking neural networks are biologically plausible and are used for biological models. In contrast, artificial neural networks have continuous values instead of spikes and are not biologically plausible, used in classification and recognition tasks. Another difference between these two is activation function; SNNs have discrete values or spikes, so these values or spikes cannot differentiate and often potentially recurrent due to the accumulation of membrane potential. Spiking Neural Networks are contenders to conquer neural computation limitations and effectively exploit real-world deep learning applications. Spiking Neural Networks are stimulated by brain mechanism [14,15,16,17] which can significantly process data in the form of spikes (discrete values) [18]. The dynamics of the SNN are to be simulated by the Leaky-integrate-and fire-neuron (LIF), which is categorized from the neuron’s inner state, called membrane potential of the neuron. This neuron’s potential accumulated from the input at a given time and produces an action potential or spike each time it crosses the specific threshold value. In recent times, there are few dedicated hardware units [19,20,21,22] that have been established for the usage of spiking neural networks. They are undertaking low power consumption for the event-based neuromorphic hardware in the context of sparse input data, specifically in deeper networks. Current works revealed that in event-based spiking models, the spiking activity and computational load had been decreased in the deep layers [23,24]. In contrast to ANNs training, training the SNNs remain a difficult task. The critical aspect of this is that there is no direct utilization of gradient-based optimization because SNNs have discontinuous activation functions. SNNs are non-differentiable; despite their non-differentiability, several implementations of backpropagation methods have been proposed in the scientific literature.

At first, all the spikes per neuron must be obliged throughout inference [25,26], then backpropagation methods can differentiate the information in the timing of neuron action potential. However, reducing the firing times for some neurons might reduce the network capacity. The second method is STDP (Spike time Dependent plasticity) that is an unsupervised learning mechanism and reward-modulated spike-time dependent plasticity for supervised learning to train deep spiking neural networks for object classification, recognition, and detection tasks. These bio-inspired learning mechanisms can effectively diminish the energy consumption in deep convolutional spiking neural networks [27,28]. For the deeper networks having higher than a few layers, such methods are not sufficient enough. The classification accuracy of ANNs trained using a backpropagation algorithm with just one hidden layer drops down drastically. The third method approximates the discontinuous spike activation function due to the spiking nonlinearity and then smoothing the SNN to be continuously differentiable. Accordingly, the backpropagation technique in this approach must be performed alongside explicit usage of activation functions [29]. The fourth method uses the Surrogate Gradient Descent; this method uses a different approach for solving the problems related to the discontinuous nonlinearity. Furthermore, they propose changes to reduce the potentially high algorithmic complications associated with training SNNs [29].The first usage of surrogate derivatives has been presented in [25]. Various types of surrogate gradient descent approaches are being developed: piece-wise linear $\beta max\left\{0,1-\left|U-\vartheta \right|\right\}$ [25,30,31], exponential function $\beta e^{\left\{-\gamma \left|U-\vartheta \right|\right\}}$ [32], rectangular $\beta sign\left\{\gamma \left|U-\vartheta \right|\right\}$ [33]. According to the observations demonstrated from the related studies, a perfect effective approach of the surrogate gradient do not exist. Rather, there is a huge dependency of performance on the internal design parameters such as β and γ.

The authors of [34] focused on the emerging deep learning models in SNNs. Their study initially described the SNNs architectures and their learning methods, such as supervised learning, unsupervised learning, and reinforcement learning. They have described each learning method in more detail and explained how these learning methods could be used in Deep SNNs. They have revised the Deep SNNs comprised of fully connected neural networks, feedforward, and presented shallow and Deep SNNs architecture over some digits and object datasets. They have also discussed the spiking deep belief networks, spiking restricted Boltzmann machines, and recurrent SNNs. Moreover, they have provided an inclusive summary comparison of the performance of new deep spiking networks. The primary purpose of their research has to make ANNs progress and spread effective and sophisticated deep SNNs. Moreover, [23] used the deep convolutional spiking neural networks using ANN to SNN conversion method on static datasets. In this work, they have used the conversion method (ANN to SNN) instead of directly train SNN that requires hundreds to thousands of time-steps while training deep SNNs. To regulate the Deep SNNs, they have proposed the “Spike Norm” method instead of employing batch normalization. The goal of using Spike Norm was to optimize the ratio of synaptic weights concerning neuron firing threshold and to minimize the loss and achieve state-of-the-art classification results; they have used synaptic weights in SNN. The previous neuron layer in the network has been scaled by a normalization term equivalent to the maximal neuron activation. They have also used the threshold-balancing method in which synaptic weights remain constant, and threshold values were set to the normalization factor. That conversion approach has utilized more time-steps and degraded the actual ANN performance when converted into SNN, which is inefficient for the neuromorphic hardware.

The authors of [35] proposed the hybrid computationally efficient training methodology for the deep Spiking Neural Networks. They have used the firing threshold, and weights of Spiking Neural Networks converted from Artificial Neural Networks for spike-based backpropagation. They have used the spike-based backpropagation for Deep SNNs for attaining the low latency and number of time-steps. Their techniques have some significant drawbacks: For the ANN to SNN conversion method, they have used approximately 2500 time-steps. Moreover, to optimize the learning variables after ANN to SNN conversion method built on spiking activity, the network has not influenced the spikes’ temporal statistics. Rather than using batch-normalization and dropout technique, they have employed the normalization element, measured just as the maximal output of the consistent convolutional or linear layer in the Spiking Neural Networks the threshold-balancing approach. Since [35] proposed the backpropagation algorithm, using the normalization factor and threshold-balancing methods, they have used time-steps above a hundred for the CIFAR-10, 100, and ImageNet dataset. More time-steps slow down the training and consume more power and energy, which is not adequate for the neuromorphic hips. Moreover, their proposed Deep SNNs did not achieve the state-of-art-art accuracies on the aforementioned datasets.

The authors of [36] revisited batch normalization and proposed a temporal batch normalization using the backpropagation through time (BNTT) method. They have presented backpropagation through time (BNTT) dissociated the learning variables in a BPTT layer, including the time interval to grab the temporal nature of spikes. The temporal nature of spikes evolving learning variables in backpropagation through time (BNTT) permits a neuron to handle its spike rate via a specific number of time-steps, allowing low-energy and low-latency training. They have showed their experiments on DVS-CIFAR-10, Tiny-ImageNet, CIFAR-10, and CIFAR-100 datasets. They have utilized the 25 to 30 time-steps on the complex datasets. By proposing the Batch-Normalization Through Time (BNTT) using Surrogate Gradient Descent on complex datasets, this method could not achieve the state-of-the-art performance on complex datasets, that is, the CIFAR-10 dataset. We have gone through all techniques mentioned above for the Deep Convolutional Spiking Neural Network using backpropagation on complex datasets. Till now, previous works did not achieve state-of-the-art performance using Deep Convolutional Spiking Neural Network.To attain higher performance and resolve the overfitting problems in Deep SNNs, we use the dropout technique using surrogate gradient descent by controlling the width γ and height β of surrogate gradient descent. Keeping these values too small and too large with the dropout does not overcome the overfitting issue. So, by recursively changing the values of γ and β, we have reached an optimal value to solve the overfitting issue. For the CIFAR-10 dataset, we could not solve the overfitting issue by adding the dropout ratios for the deep SNN models. So, we set an intermediate value of γ and β (i.e., 30, 2) in deep SNNs to solve the overfitting issue and attained higher performance.

In our knowledge, previous works used surrogate gradient descent backpropagation technique to train deep convolutional spiking neural networks directly [37]. By tuning surrogate gradient descent and using batch normalization, they solved the vanishing or exploding gradient problems. This problem occurs by increasing the number of neurons; however, their works have not achieved the best results on some datasets such as CIFAR-10 and SVHN. In this work, we encounter an overfitting problem in deeper layers. We use the dropout [38] technique with the surrogate gradient descent to regularize the deep SNN during training to solve the overfitting issue. To train deep SNNs, we have used the dropout technique by controlling the width γ and height β of surrogate gradient descent. Keeping these values too small and too large with the dropout does not overcome the overfitting issue. So, by recursively changing the values of γ and β, we have reached an optimal value to solve the overfitting issue. For the CIFAR-10 dataset, at first, we could not solve the overfitting issue by adding the dropout for the deep SNN models. So, we set an intermediate value of γ and β (i.e., 30, 2) in deep SNNs to solve the overfitting issue and attained better performance. Training SNNs with backpropagation techniques, i.e; backpropagation through time, spike-based backpropagation, requires more time-steps, and some previous works achieved good performance on static and neuromorphic datasets. However, these backpropagation approaches with more time-steps take a-lot of time for training SNN that have not been efficient for neuromorphic hardware.Moreover, some authors managed to achieve good results on some datasets by converting the ANN to SNN [23] methods; however, this converted method needs a hundred to thousand time-steps to attain the best performance. To achieve the best classification results on different datasets and reduce the processing time, we have used fewer time-steps with surrogate gradient descent for deep convolutional spiking neural networks. For the classification and recognition tasks, training SNNs on few datasets such as CIFAR-10, SVHN [37,39] is complex with deeper layers, and it takes much time to train.

The authors of [40] proposed a novel SparkXD framework to attain robust and energy-effective Spiking Neural Network (SNN) inference below estimated DRAM over error-aware DRAM mapping and error-tolerate analysis. That method lessened the DRAM energy by 40% on average; on the other hand, managing the accuracy inside 1% of the standard Spiking Neural Network (SNN). The proposed method by [40] has done their experiment on the shallow network for the MNIST dataset. On the other hand, [41] has also proposed an optimization framework for encoder, model, and architecture design of FPGA-based neuromorphic hardware. They also deployed their shallow SNNs for the datasets such as MNIST, Fashion MNIST, Spoken Arabic Digits, and so forth, to compare the inference latency and power. The purpose of their study has to make the SNN consuming low power and energy for the shallow networks over datasets such as MNIST, Fashion MNIST, and so forth. However, in our work, we are more concerned with the deep SNNs and their classification performance and processing time. So, all of our experiments have conducted on MNIST, CIFAR-10, KITTI, SVHN, and License Plate dataset using shallow and Deep SNNs. The authors have not used any embedded platform to evaluate the processing time and inference accuracy for the Deep Convolutional Spiking Neural Networks. This work uses the NVIDIA JETSON TX2 board to deploy all the SNNs and compare the processing time, inference accuracy w.r.t PC.Our goal of using an embedded platform is to show the feasibility of proposed deep convolutional spiking neural networks. We want to understand; such SNN architectures could be implemented with ANN, like structure or not. In this research work, we discuss the feasibility of explicitly training deep convolutional spiking neural networks. To accomplish this, a surrogate gradient descent, as proposed in [29] has been used. Moreover, we exploit the main idea of effective deep ANN models with LeNet5 [42], VGG [43], and ResNet [44] for optimized construction of deep convolutional spiking neural network architectures.The major contributions of this work are defined as follows:

1. This work proposes various Deep Convolutional SNNs to classify the privately acquired Korean License plate and KITTI dataset. Moreover, this work proposes deep Convolutional SNNs for MNIST, SVHN, and CIFAR-10 datasets.
2. To encounter the overfitting issue during training deep SNNs, this work uses SNN based dropout technique [39] by altering the scale, width, and height parameters of surrogate gradient descent.
3. This work evaluates the performances of the SNNs on both the embedded platform (NVIDIA JETSON TX2) and PC in the context of the processing time and inference accuracy w.r.t various datasets (both public and private).
4. This work also uses the fewer orders of magnitude in terms of inference time steps (8,10,20) with surrogate gradient descent on customized Deep SNNs to achieve the best results, thereby minimizing the inference energy and time.

## 2. Spiking Neuron Model

### 2.1. Leaky-Integrate-and-Fire (LIF) Neurons

**Figure 1:**
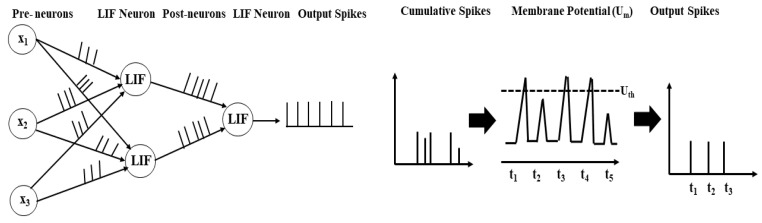
The spike generation mechanism of neurons in the spiking neuron model (LIF). The pre-synaptic neurons or spikes $x_{i}\left(t-t_{k}\right)$ are controlled by interconnecting synaptic weights $\left(w_{i}\right)$ to generate post-synaptic neurons or spikes. The total input values combine into post-synaptic membrane potential $\left(U_{m}\right)$ that leaks exponentially with time and with a time constant $\left(\tau_{m}\right)$. If the neuron’s membrane potential of the pre-synaptic neurons crosses a specified threshold $\left(U_{th}\right)$, post-synaptic spikes are generated and then rearranges its membrane potential to the starting value.

Figure 1 shows the overall spike generation mechanism in the LIF neuron model. LIF neuron describes the internal structure of neuron along with the shift in membrane potential of the neuron and spike generation [45]. The dynamics of SNNs are to be simulated by two essential computational elements i.e., LIF neurons and interlinked synapses. The threshold mechanism of leaky-integrate-and-fire neurons is defined as:

(1) $$\tau_{m}\frac{dU_{m}\left(t\right)}{dt}=-U_{m}\left(t\right)+i\left(t\right),$$

where $U_{m}\left(t\right)$ represents the membrane potential of the input and output neuron and $\tau_{m}$ shows the decay term for the membrane potential. The input synaptic current, $i\left(t\right)$ is the weighted sum of pre-synaptic neurons over a given time-steps.

(2) $$i\left(t\right)=\sum_{i=1}^{a^{l}}\left(w_{i}\sum_{k}x_{i}\left(t-t_{k}\right)\right),$$

where $a^{l}$ shows the amount of input neurons, $w_{i}$ represents the synaptic weight connecting with $i^{th}$ input neurons to output neurons. $x_{i}\left(t-t_{k}\right)$ is the spike occurrence from the $i^{th}$ input neuron over time $t_{k}$, that is expressed as a Kronecker delta function shown below:

(3) $$x_{i}\left(t-t_{k}\right)=\left\{\begin{array}{cc} 1, & \mathrm{if}\;t=t_{k}\\ 0, & \mathrm{otherwise}, \end{array}\right.$$

where $t_{k}$ is the time of incoming spike occurrence, denoted as $x_{i}\left(t-t_{k}\right)$.

Figure 1 shows the LIF neuronal mechanism. The input neurons or spikes $x_{i}\left(t-t_{k}\right)$ are controlled by interconnecting synaptic weights $w_{i}$ to generate output neurons or spikes. The input neuron current is combined into the output membrane potential $U_{m}\left(t\right)$ that leaks with time and time constant $\tau_{m}$. If the input neurons’ membrane potential crosses a defined threshold $U_{th}$, output spikes are generated and then rearranges its membrane potential to the starting position.

### 2.2. Deep Convolutional Spiking Neural Networks (DCSNNs)

#### Spiking Convolutional and Pooling Operation

This work establishes a training method for deep convolutional spiking neural network architectures consisting of the input neurons preceded by some hidden layer that comprises center neurons and the final classification layer. At first, the input values from the given images are converted into spike trains using the Poisson-distribution. The possibility of generating an action potential or spike depends on the strength of the pixels. Hidden layers consist of numerous convolutional and average-pooling layers, which are sorted alternately in the SNN model. These layers draw out the complex and straightforward information from the given input image. Finally, the last pooling layer’s spikes are merged to produce a unidimensional vector input for the linear layers (fully connected layer) used for the final classification. The learning process of image features occurs between the convolutional and fully connected layers, while the pooling layers are specified initially.

**Figure 2:**
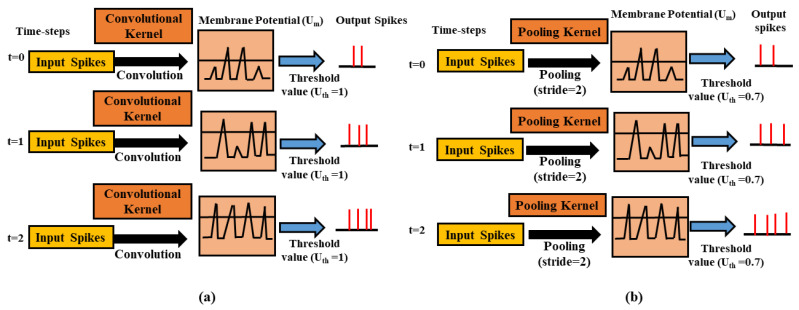
Demonstration of convolutional (**a**) and average-pooling method, (**b**) above three different time-steps. At a given time interval, the pre-neurons or spikes are combined with convolutional or pooling kernels to measure the input current, which is then combined into the neuron’s membrane potential, $U_{m}\left(t\right)$ at each time-step. If the membrane potential of that neuron $U_{m}\left(t\right)$ is greater than a specified threshold value $U_{th}$, the spikes are produced, and $U_{m}\left(t\right)$ goes to its initial value, i.e., 0. Alternatively, over the next time step, $U_{m}\left(t\right)$ is assumed to be residual while leakage throughout the current time-step.

Figure 2 illustrates the basic operative example of a convolutional layer comprising some LIF neurons above three different time-steps. During training, each input spike train combines with the kernels in convolutional layers to calculate its input current. That input current is then combined into the neuron’s membrane potential, $U_{m}\left(t\right)$ at each time-step. If the membrane potential of the neuron $U_{m}\left(t\right)$ is more significant than a specified threshold value $U_{th}$, the neuron spikes, and $U_{m}\left(t\right),$ goes to its initial value, i.e., 0. Alternatively, over the next time-step, $U_{m}\left(t\right)$ is assumed to be residual while leakage throughout the defined time-steps.

Figure 2 illustrates the basic function of the average-pooling method, which lessens the dimensions of the preceding convolutional layer while preserving spatial statistics. We perform the spatial-pooling operation in several ways in an artificial neural network. In the spiking neural networks pooling operation can be used as e.g., max-pooling [24] and average-pooling [46,47]. Owing to its easiness, we use average pooling in this work. In SNNs, an auxiliary threshold is used after averaging the neurons to produce a post or output spike. In Figure 2, the threshold value for the average-pooling layer must be set carefully so that the spike distribution does not interrupt due to the pooling layer. There is a chance of too many spikes because of the low threshold value in the average-pooling layer that can cause loss of spatial information collected from the preceding layer. When the threshold value is large enough, spikes distribution in the deeper layers will not be adequate.

### 2.3. Deep Convolutional Spiking Neural Networks (DSCSNNs): Spiking ResNet and VGG

**Figure 3:**
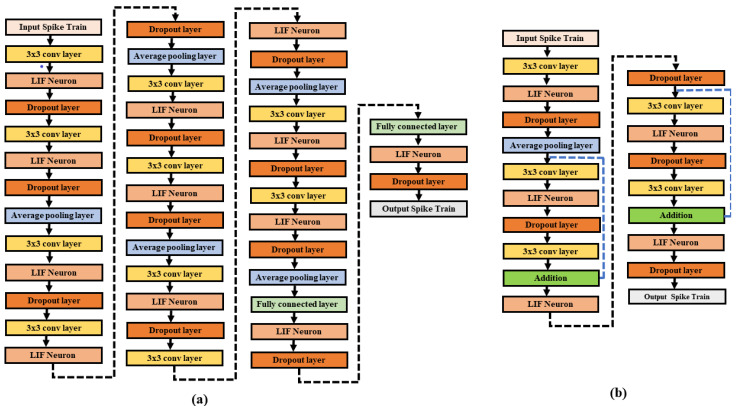
Network Architectures: (**a**) VGG-13 (**b**) ResNet-6.

Deep neural networks play a significant role in learning complex input patterns from the given images to efficiently learn hierarchical illustrations. Following the effectiveness of deep models, we examine the most common deep neural network architectures such as ResNet [44], and VGG [43]. VGG is the first deep neural network that uses 3×3 small kernels in the network. Using 3×3 small filters in a spiking VGG network enables an efficient accumulation of convolutional layers while reducing the number of model variables in deep models. In this research work, we are dealing with deep convolutional spiking neural networks (DCSNNs) using spiking VGG and spiking residual networks (ResNet). In the Spiking VGG-13 model, we have a stack of convolutional layers, succeeded by a LIF layer, average-pooling layer, dropout layer, and fully connected layer for classification. Figure 3 shows the Spiking VGG-13 model consisting of 10 convolutional layers, each followed by a leaky-integrated-and-fire layer that consists of an activation function, a dropout layer that is used to avoid overfitting, an average-pooling layer that diminishes the size of the previous layer while retaining spatial statistics and the fully connected layer for final classification.

Next, the ResNet-6 model [44] has developed skip connections across the network that has tremendous success in allowing the efficient training of deep networks. The degradation problem is observed in the ResNet model [44] during the training process. This problem occurs by increasing the network’s depth and is solved using SNN based ResNet-6 architecture shown in Figure 3 consisting of leaky-integrate-and-fire neuron layer, dropout layer, convolutional layers, and an output layer. To reduce the degradation issue, we utilize the idea of skip connections to create SNN based ResNet-6 model. When the input and output feature characteristics are equal, then the residual connection includes identity mapping, and when the input and output feature characteristics vary, then skip connection or residual connection contains 1×1 convolutional kernels. The output spikes of the last convolutional layer (residual) and output spikes of non-residual connections are accumulated to the last leaky-integrate-and-fire neuron’s membrane potential layer to produce post spikes from the ResNet-6 model.

The dropout layer is used before the pooling layer for the spiking VGG-13 and spiking ResNet-6 model to avoid overfitting. Dropout is only used throughout the training process and then when assessing the model’s efficiency during inference. Inside the spiking VGG and ResNet model, we have an average-pooling layer that decreases the earlier convolutional layer dimensions while preserving spatial statistics. Finally, we have a fully connected layer containing numerous neurons as the total number of classes for a classification task. The fully connected layer contains synaptic weights. The spikes produced in the preceding layer (pooling layer) are added to make a unidimensional vector for classification at the output layer.

## 3. Training Deep Spiking Neural Networks

### 3.1. Surrogate Gradient Descent

At first, assuming a single neuron and examine how to train the neuron for generating a defined spike train $X_{gt}\left(t\right)$ for a specified stimulus [37]. The author defined the error function as follows: The energy is taken as an integral of a time period equal to that of the error current $X_{err}\left(t\right)=X\left(t\right)-X_{gt}\left(t\right)$ raised by the membrane potential of the neuron $U_{m}\left(t\right)$. Therefore, the loss function is defined as:

(4) $$\begin{array}{cc} E_{loss} & =\int_{0}^{T}X_{err}\left(t\right)U_{m}\left(t\right)d\left(t\right)\\ \; & =\int_{0}^{T}\left(X\left(t\right)-X_{gt}\left(t\right)\right)U_{m}\left(t\right)d\left(t\right), \end{array}$$

where *T* is representing the total spike train duration, such loss function has no issues with the non-differentiability of the spiking nonlinearity since the gradient of the output X(t) is null all around and ignored [48], with the function $\sigma \left(U_{m}\right)=U_{m}$. The loss function defined in (4) has some necessary characteristics: the loss value gets reduced with the growing membrane potential when the output spike pulse is absent $\left(X-X_{gt}<0\right)$ hence allowing the output spike pulse to appear.On the other hand, the loss grows with the decline in membrane potential when the output spike pulse does not present $\left(X-X_{gt}>0\right)$. The disadvantage of this kind of loss function is that it can obtain mutually positive and negative values, and values near zero can be found when the membrane potential is close to zero even if output pulses fluctuate from those of the chosen ones. However, the ideal values for the weights equivalent to the gradient loss are zero. Similarly, [37] defined a more general loss with the van Rossum distance.

(5) $$E_{loss}=\int_{0}^{T}\left[\left(a\times X\right)\left(t\right)-\left(a\times X_{gt}\right)\left(t\right)\right]\left(a\times U_{m}\right)\left(t\right)d\left(t\right)$$

(6) $$\left(a\times V\right)\left(t\right)=\int_{0}^{t}a\left(t-t^{{}^{\prime }}\right)V\left(t^{{}^{\prime }}\right)dt^{{}^{\prime }},$$

where (6) represents a convolution. For example, If the number of spike pulses is greater than a defined threshold, only one neuron can be used for classification purposes. Similarly, in (4), [48] has taken the convolutional kernel $a\left(t\right)=1$ for the entire interval *T* and presented a below loss function for classification purposes:

(7) $$E_{loss}=\left[\Theta \left(O-O_{g}t\right)\right]\int_{0}^{T}U_{m}\left(t\right)d\left(t\right),O=\int_{0}^{T}X\left(t\right)d\left(t\right),$$

where the symbol Θ represents a Heaviside step function, $O_{gt}=0,1$ indicates the ground-truth labels. The gradient of the loss function in (4) can be found subsequently:

(8) $$E_{loss}^{\prime }=\frac{1}{2}\int_{0}^{T}{\left[X\left(t\right)-X_{gt}\left(t\right)\right]}^{2}dt.$$

As an alternative to (4) and

(9) $$E_{loss}^{\prime }=\frac{1}{2}{\left[\Theta \left(O\right)-O_{gt}\right]}^{2},$$

rather than (7) in conjunction with a modified rule

(10) ∇X⟶∇U.

Such that, the gradient method of non-differentiable spikes is swap by a different surrogate gradient method [29].

### 3.2. Surrogate Gradient Descent for Deeper SNNs

The training SNNs with backpropagation is to exchange the gradient of a non-differentiable spike with an alternative surrogate gradient [29]. The straightforward substitution ∇X⟶∇U but, when applied to deeper layers of SNN, this form of gradient leads to problems. In this case, when the hidden neuron’s membrane potential is near zero states, gradient descent operation leads the potential to rise or decline of virtually equivalent probabilities, and the membrane potential stays to zero for an extended period. To prevent this issue, [37] change the substitution rule as:

(11) $$\nabla X⟶f(U_{m})\nabla U,$$

when the membrane potential is near to zero, then $f(U_{m})$ becomes lesser: f(0) is less than $f(U_{th})$. Mathematical results demonstrate that the real method of the function $f(U_{m})$ is not significant. The authors of [37] modified the substitution rule as:

(12) $$f\left(U_{m}\right)=b{\left\{1+\left[c\left(U_{m}-U_{th}\right)\right]\right\}}^{-2}.$$

**Figure 4:**
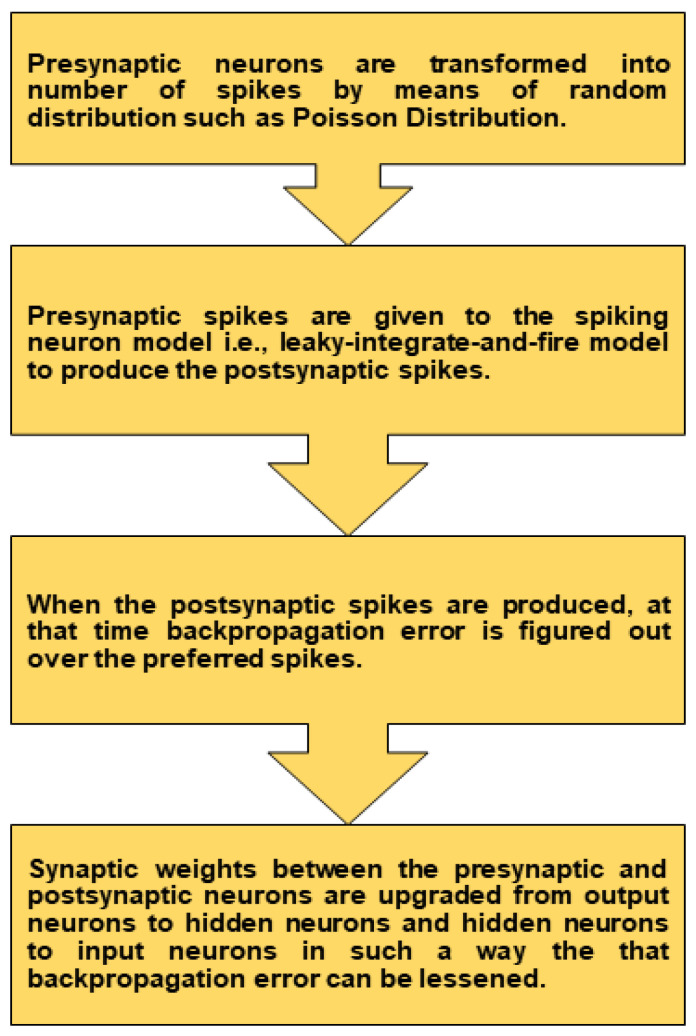
Flow diagram of the training process.

Here, (b=1) represents a hyper-parameter that shows the surrogate gradient size, and *c* shows the gradient thickness. As seen in the above section, the term c=0 is not enough for deeper SNNs. In contrast, big values of *c* make the surrogate gradient often quite small apart from extreme situations where the membrane potential $U_{m}$ is near the threshold $U_{th}$ value. Therefore, an ideal intermediate value of *c* should be assumed to exist. Remember, we consider the gradients of spikes $x_{k}^{t}$ to be zero along with hard reset SNN neurons. The earlier values of the membrane potential $u_{m_{k}}$ do not affect the membrane potential gradient after resetting; the history of the neuron’s membrane potential is lost. Figure 4 shows the overall training process of the SNNs.

### 3.3. Dropout in Deep Spiking Neural Network

Dropout [38] is a common technique in artificial neural networks to avoid overfitting during the training process. This method arbitrarily detaches components with a given probability (p) to prevent overfitting and co-adapting to the training data. Previous works [49,50,51] have inspected the biological perceptions of how synaptic stochasticity comes up with dropout-like advantages in spiking neural networks. In this research work, we use the idea of the dropout method to regularize the deep convolutional spiking neural networks efficiently. The dropout approach is only used in the training phase but is not used to test the network efficiency during inference.

However, in SNNs, dropout [39] is used in a different way than ANNs. In ANNs, each single training epoch has numerous repetitions of mini-batches. In every repetition, arbitrarily chosen components (including a dropout rate of *p*) are separate from the model and at the same time measuring its subsequent probability ($\frac{1}{1-p}$). In spiking neural networks, each repetition has numerous forward propagations depending on the spike train duration. An output error term is to be backpropagated and change the model parameters at the final time steps.

In the training phase, to make the dropout efficient, it must be a guarantee that the component associated with each repetition of batch information does not alter; thus, the DNN (deep neural network) is comprised of similar arbitrarily subcategories of components throughout the forward propagation in the single repetition. Conversely, during every time-step, if the units or components arbitrarily link with the result of dropout, then it will be added up through the whole forward propagation time steps in all repetition. At that time, the dropout result would disappear when the output error function is backpropagated, and then the model values will be upgraded only at the final time interval. Therefore, we require that the set of arbitrarily associated components be maintained for the whole time in each repetition. In this work, we utilize the spiking neural network form of the dropout method along with the likelihood (*p*) of neglecting ratios from 0.2 to 0.25. Due to sparser activations in spiking neural networks forward propagations instead of ANNs, the ideal value of dropout for a spiking neural network needs less than a traditional ANN dropout ratio (i.e., 0.5).

## 4. Experiments and Results

This section illustrates the efficiency of our proposed deep convolutional spiking neural networks (DCSNNs) using the backpropagation technique, that is, surrogate gradient descent. First, we explain the experimental structure and background. For the observations, we have built a customized simulation structure using Pytorch’s [52] deep learning library to test our presented spiking neural network training algorithms.

Our proposed eep convolutional spiking neural networks (DCSNNs) comprise leaky-integrate-and-fire neurons (with neuron firing threshold value of 1) in which input and output neurons are linked through adjustable weights, i.e., synapses. Initially, we adjusted the synaptic weights by using random distribution i.e., Gaussian distribution with zero-mean and standard deviation of the square root $(\;\frac{u}{i^{1}})\;$ where $i^{l}$ pre-synaptic neurons as proposed in [53]. The constant *u* value varies depending upon the form of the model structure. For instance, we have taken u=2 for non-residual connection and u=1 for residual connection in ResNet-6 model. For the public and private dataset, we train our proposed models for 100th, 125th training epochs with a mini-batch SGD optimizer that decreases its learning rate at 30th, 60th and 115th epochs. Table 1 shows the execution details.

### 4.1. Private Data Set

**Figure 5:**
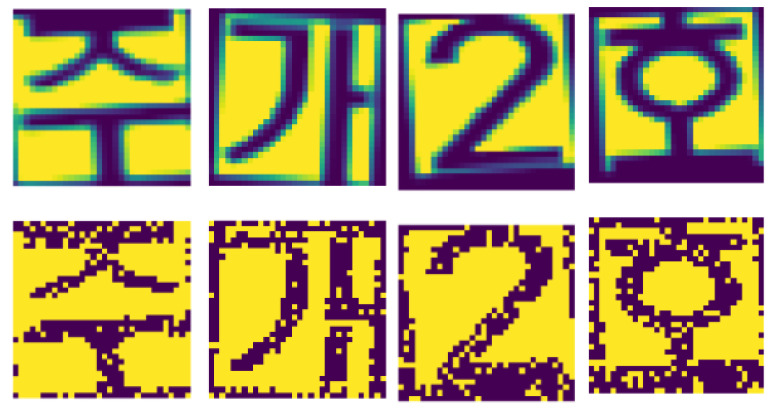
Images from the License plate dataset are converted into spikes using Poisson distribution. The upper images show original images, and the below images represent the spike version of original images.

We demonstrate our proposed ResNet-6 and VGG-6 architecture effectiveness on the license plate dataset and KITTI dataset. The License plate dataset consists of numbers from 1–9, some Korean characters whose image sizes are 32 by 32. Each digit and character are cropped from the license plate data and use data augmentation techniques such as translation, rotation, shear, and random horizontal flipped. For license plate data, each input pixel intensity is transformed into several spikes using Poisson-distribution with an appropriate firing rate. Figure 5 shows the original and spike version of the license plate dataset. If an image pixel intensity is higher, more spikes are produced, while if the pixel intensity of an image is lower, fewer spikes are produced. Pixels with greater value have more spikes and be “on”. The middle-value pixels, relative to their density, are “on" and “off” time by time, but the low-density pixels with low density are always “off”.

**Figure 6:**
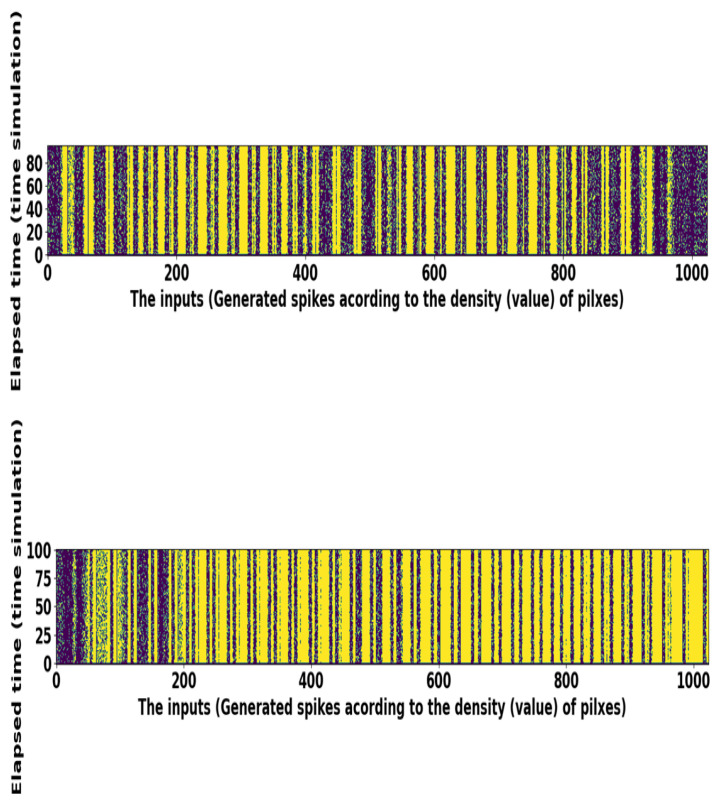
Raster plots of random spike trains taken from the license plate dataset.

This problem is exemplified in the spike train raster-plot of 1024 neurons corresponding to the license plate image pixel values. Most of the time, the "on" pixel values are situated in the middle of images; therefore, we can see some darker spike trains in the center in Figure 6. Precisely, for a given time window, the input pixel values are associated with a distribution, namely a uniform random number between 0 and 1. The description of the license plate dataset is presented in Table 2. We also use the KITTI dataset to classify six different classes: person, pedestrian, truck, car, bicycle, and motorcycle. Each image is cropped and resized to 32×32 size. We implement the SNN based VGG-6 model over the KITTI dataset and attain good classification performance. The details of the KITTI dataset demonstrates in Table 2.

### 4.2. Public Data Set

**Figure 7:**
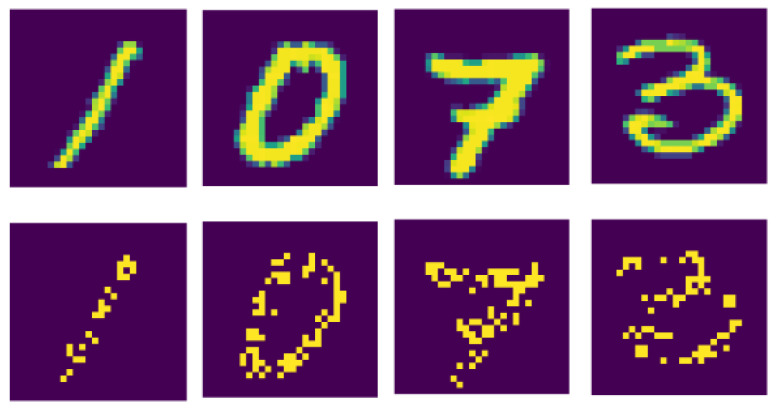
Original images of the MNIST dataset are converted into spikes using Poisson distribution. The upper images show original images, and the below images represent the spike version of original images.

We also show the efficiency of our presented deep convolutional spiking neural networks on some benchmark datasets MNIST [42], Street view house number [54], and CIFAR-10 [55]. The MNIST dataset has gray-scale images of size 28×28. For the MNIST dataset, each input pixel intensity is transformed into several spikes using Poisson-distribution with an appropriate firing rate. Figure 7 shows the original and spike version of the MNIST dataset.

**Figure 8:**
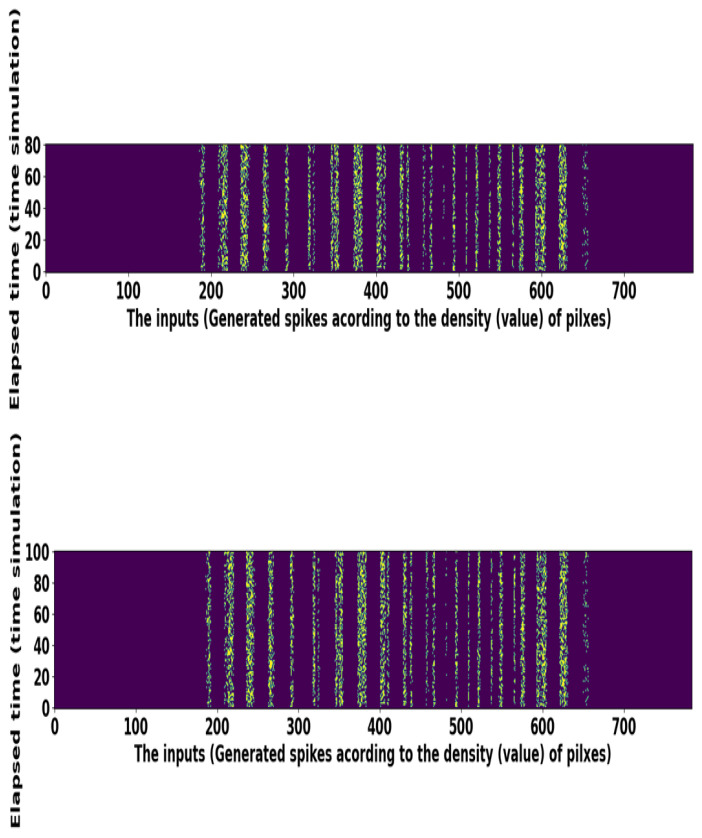
Raster plots of random spike trains taken from the MNIST dataset.

Unlike the license plate dataset, for MNIST, if the pixel intensity of an image is higher, more spikes are produced, while if the pixel intensity of an image is lower, fewer spikes are produced. Pixels with greater value have more spikes and are “on.” The middle-value pixels, relative to their density, are “on” and “off” time by time, but the low-density pixels with low density are always “off.” This problem is demonstrated in the spike train raster-plot of 784 neurons corresponding to several image pixel values. The “on” pixel values are sometimes positioned in the center of images; therefore, we can see some darker spike trains in the center in Figure 8. The CIFAR-10 and SVHN dataset have colored images of size 32×32. Each image in MNIST, CIFAR-10, and SVHN is transformed into a sequence of spikes using Poisson distribution. We have applied data augmentation techniques for the CIFAR-10 and SVHN datasets, that is, random horizontal flip and random cropping. After applying the data augmentation technique, then we converted each image into several spike trains. Such image pixel intensities are standardized to specify mean and standard deviation (0 and 1). We then calculate the pixel intensities of input images and bring them between −1 to 1 to show the entire distribution of input pixel depictions. After that, the standardized input images are transformed into spikes by using Poisson distribution. Description of these vision datasets is presented in Table 2.

#### 4.2.1. Configurations of Network

We use numerous deep convolutional spiking neural networks (DCSNNs) for the private and benchmark datasets. For the license plate dataset, we used ResNet-6 and ResNet-7 models. In the ResNet-6 model, several convolutional layers convolve with pooling layer filters or kernels that have the stride of 2. In the VGG-6 model, six layers, that is, four convolutional layers and two fully connected layers for the KITTI dataset, have been applied. For the MNIST dataset, we performed a four-layer spiking neural network comprising two convolutional layers and two fully connected layers.

Table 3 states the comprehensive network details of a License plate, MNIST, and the KITTI dataset. Deep convolutional spiking neural networks as VGG-7, VGG-8, VGG-11, and VGG-13 consisting of convolutional, average-pooling, and fully connected layers are used on the SVHN and CIFAR-10 dataset. Table 4 states the overall network explanation of SVHN and CIFAR-10.

#### 4.2.2. Comparison of Classification Performance with Related Works

In this section, we compare classification performance with the state-of-the-art works. First, we have compared the classification performance achieved by our proposed deep convolutional spiking neural networks (DCSNNs) w.r.t the performance of supervised, unsupervised, converted ANN to SNN methods and directly trained SNNs. In the recent research, authors have used Deep SNNs for the classification of digits (MNIST) and objects (CIFAR-10) datasets. Previously, authors have used supervised [29,56,57,58] and unsupervised [59,60,61,62,63] learning methods for training Deep SNNs. In unsupervised learning, refs. [59,60,61,62,63] has used a new learning mechanism called Spike-Time-Dependent Plasticity (STDP). That learning method is used for the classification and recognition tasks with more time-steps above 100, and the authors have attained a good performance using shallow SNNs. Using STDP [27] has attained 98.40% accuracy on the MNIST dataset below state-of-the-art performance. However, the STDP learning mechanism is limited to shallow networks such as three and four layers and could not perform well for the Deep SNNs.

Moreover, some works [27,64,65] have combined both these unsupervised and supervised-based learning methods to train the SNN and attained the highest results on both approaches using shallow SNNs with time-steps 100 and 200. However, these approaches have also been limited to shallow models and could not be functioned on large-sized networks. These approaches achieved below 98% accuracy on the MNIST dataset and 90% accuracy on the CIFAR-10 dataset. These approaches are far away from the state-of-the-art classification and recognition inference results.

Few authors put forward ANN to SNN conversion methods for the training of deep SNNs [23,24,47,66] and attained approximately 99.10% classification accuracy on the MNIST dataset. Till now, ANN to SNN conversion methods [23,24,67] accomplished the highest inference results on the CIFAR-10 dataset, that is, 93.63%. However, these converted methods need hundreds to thousands of time-steps to train deep convolutional SNNs. This ANN to SNN degrade the actual ANN performance when converted into SNN, and training is prolonged for the deep SNNs.

In this work, our approach of directly training SNNs achieved state-of-the-art inference results using surrogate gradient descent over MNIST and CIFAR-10 datasets about 99.66% and 91.58% with effective processing time. Moreover, we have used fewer time-steps for the fast and energy-efficient SNNs using surrogate gradient descent and achieved the highest classification results till now. Table 5 demonstrates the associated classification results using the proposed approach against other methods. Table 6 shows optimized parameters used in our work compared with the recent works over MNIST and CIFAR-10 dataset. For the MNIST dataset, we achieved good accuracy as compared to the related works. The CIFAR-10 dataset analyzes the most challenging to train directly with SNNs, and we attained the best accuracy compared to related works. We also evaluated the preceding best SNN training performance in the related works with proposed deeper SNN models. Later by adjusting the weights, we trained the SNNs using surrogate gradient descent with input images that must be converted into spikes using Poisson distribution.

**Figure 9:**
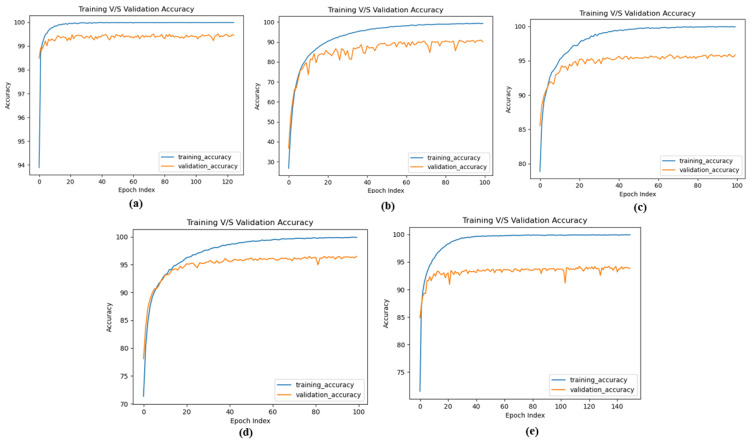
Training and Validation curves: (**a**) MNIST (**b**) CIFAR-10 (**c**) KITTI (**d**) Korean License Plate (**e**) SVHN.

Our analysis of the MNIST dataset produces a classification result of 99.66%, which is the highest associated performance compared to other SNN training methods and indistinguishable from other ANN to SNN converted approaches. Furthermore, we attain the best inference results on the SVHN dataset, about 94.01% for VGG-8 and 93.7% VGG-9 based SNN models. For the license plate dataset, which has not been listed in the literature, we achieve approximately 96.46% and 93.7% classification inference results for ResNet-6 and ResNet-7 SNN models, respectively. Finally, for the CIFAR-10 dataset, a very challenging dataset to train directly using spiking neural networks, we used VGG-11 and VGG-13 SNN based models and reached around 91.25% and 91.58% inference results, respectively. All-inclusive, our presented Deep Convolutional Spiking Neural Network(DCSNN) accomplishes the best inference results for SNN based ResNet and VGG models. Figure 9 shows the training and validation curves of MNIST, CIFAR-10, KITTI, Korean License plate and SVHN datasets.

#### 4.2.3. Classification Results by Increasing the Number of Network Layers

We have trained different models for the License plate, KITTI, SVHN, and CIFAR-10 datasets to investigate SNN performance with surrogate gradient descent by increasing the model layers. We began with the four-layer SNN model that consists of two convolutional layers and two fully connected layers for the license plate dataset. By performing experiments on the license plate dataset with four-layer SNN, we have encountered an overfitting issue. Then, we changed our four-layer SNN network to six layers as the VGG-6 model. On the SNN based VGG-6 model, we achieved an inference accuracy of around 91.45%. After that, we proceeded with our experiments by making SNN based ResNet model. So, we implemented ResNet-6 and ResNet-7 models on license plate datasets and achieved an inference accuracy of nearly 96.46% and 93.68%, respectively. For the KITTI dataset, we started experiments from a four-layer network, and finally, the SNN based VGG-6 model performed well and achieved the highest results. For the SVHN, we followed the same network structure as four-layer SNN.

**Figure 10:**
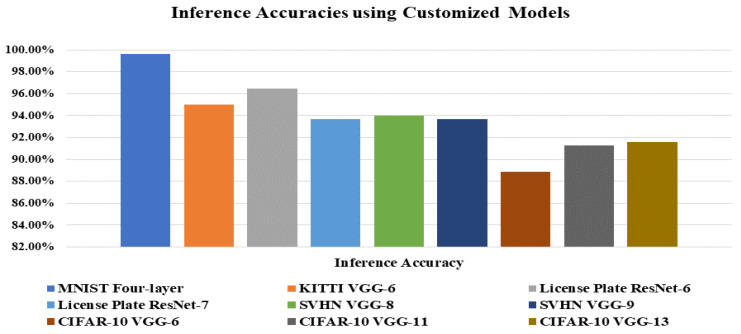
Inference accuracy performance, along with different DCSNN models and datasets.

At first, for four-layer SNN, we attained an inference accuracy of 89.96%. Our model performance has improved by increasing the network depth from four layers to eight and nine-layer. We implemented VGG-8 and VGG-9 based SNN model on SVHN datasets and reached an inference accuracy of approximately 94.01% and 93.68%, respectively. At last, we proceeded with the same experiments on the CIFAR-10 dataset. We managed experiments on CIFAR-10 datasets with SNN based VGG-6 model, reached an inference accuracy of 86%. Then we started to increase the layers of the SNN based VGG-7, VGG-8, VGG-11, VGG-13. So, for VGG-7, VGG-8, VGG-11, VGG-13, we reached an accuracy of around 88.16%, 90.56%, 91.25%, and 91.58%, respectively. Figure 10 shows the inference accuracy and proposed SNNs architectures over private and public datasets.

#### 4.2.4. Classification Results and Number of Inference Time-Steps

**Figure 11:**
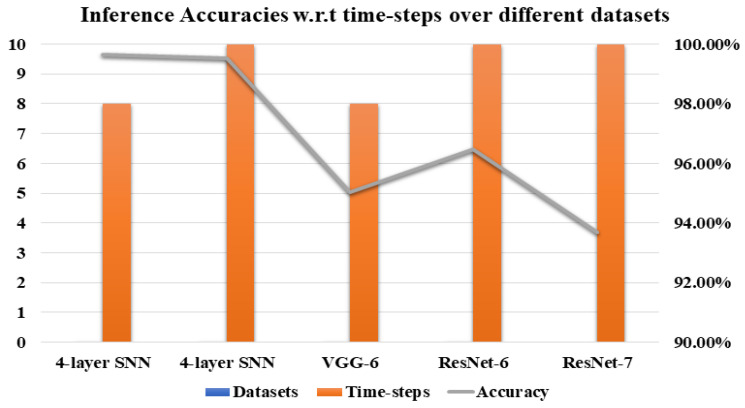
Classfication Performance w.r.t time-steps over MNIST, KITTI and License Plate dataset.

To check the SNNs classification performance, different time-steps (8,10,15,20) have been used. In all our experiments, we have observed that our network’s performance varies by increasing or decreasing the number of time-steps. First, SNN based four-layer and VGG-6 have trained using the number of time-steps, that is, 8 and 10. We have seen that managing a smaller number of time-steps increases inference accuracy. Figure 11 clearly illustrates that by keeping smaller number of time-steps increases the inference performance. It shows the inference accuracies over MNIST, KITTI, and License Plate datasets using time-steps 8 and 10. In the case of the MNIST and KITTI dataset, we can see in the Figure 11, the classification accuracy reaches above 99% for time-steps 8 and 10. In the case of deeper models for the License plate and SVHN dataset, 10 and 15 time-steps have been adopted. For 20 time-steps, inference accuracy increases, but with the rise in time-steps from 20 to 30, the inference accuracy substantially declined.

**Figure 12:**
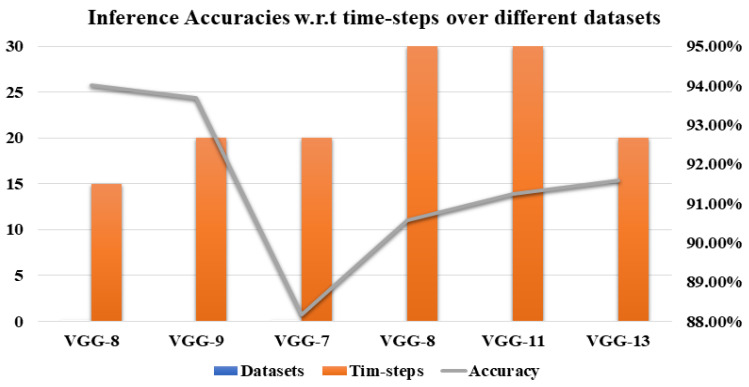
Classfication Performance w.r.t time-steps over CIFAR-10 and SVHN dataset.

Likewise, for the CIFAR-10 dataset, we have considered 10 and 20 time-steps for SNN based VGG-13 model where the inference performance is degraded. Then by decreasing the time-steps from 20 to 15, the SNN has attained the best accuracy. In the literature, studies have used [23,47,66] ANN to SNN conversion methods, which requires hundreds to thousands of times-steps during training. Nevertheless, these conversion methods are hard to train and often degrade classification performance during training. We have adapted fewer time-steps with surrogate gradient descent to train customized SNNs that provide the best classification results in this work.It can be shown in Figure 12 that by increasing the number of time-steps for complex datasets such as CIFAR-10, SVHN decreases the classification performance. We use 20 time-steps for the SVHN dataset, which increases the inference accuracy and attains 95% inference accuracy; however, using the same time-steps for the CIFAR-10 dataset decreases the performance. In the Figure 12, for the CIFAR-10 dataset, we use time-steps 10 and 15 using VGG-11 and VGG-13 models, and the classification performance rises and achieves approximately 92% inference accuracy.

## 5. Performance Evaluation on Embedded Platform

This work used an embedded platform to compare the processing time and inference accuracy of SNNs and ANNs on both PC and NVIDIA JETSON TX2. We have used all the same proposed SNNs for ANNs to check the processing time and inference accuracy. All trained DCSNNs and ANNs models have been deployed on an embedded platform. First, we have deployed a shallow model of SNNs and ANNs and calculated the processing time (milliseconds) and inference accuracy. We have seen that for the shallow model, SNNs outperform than ANNs in terms of accuracy. We have then deployed trained DCSNN and ANNs models on the board and measured the processing time and inference accuracy. Deep models consume more resources in terms of inference accuracy and processing time for SNNs. Compared with ANNs, SNNs performance has declined in terms of accuracy and processing time for deeper models. Table 7 has listed the processing time and inference accuracy of both SNNs and ANNs on the PC and an embedded platform w.r.t diverse datasets.

In Table 7, for each dataset, the processing time for the PC and the embedded platform is calculated as the average processing time over the entire test batch. In the case of MNIST, we have seen that SNN performance is increased than ANN regarding accuracy. However, processing time (per image) on PC and board for SNN is still higher than ANN. For the KITTI dataset, the PC’s processing time is approximately four times faster than the embedded platform, and accuracy has improved by deploying the SNN model. However, ANNs outperform in terms of accuracy and processing time (per image) than SNNs on an embedded platform. For the License Plate dataset, in the case of SNNs, we examine that the accuracy and processing time (per image) of SNNs outperforms than ANNs. Following the same experiments of SNNs and ANNs, for the SVHN dataset, we have investigated that accuracy and processing time (per image) is more significant for SNNs than ANNs on both PC and an embedded platform. Subsequently, for the CIFAR-10 dataset, all the SNN and ANN-based VGG models have deployed and calculated the processing time and inference accuracy. We can see that the processing time for VGG-11, VGG-13 models for ANNs on the PC and the NVIDIA board is higher than the SNNs. NVIDIA JETSON TX2 has been used to validate the accuracies and processing time over different datasets for ANNs and SNNs.

Our goal of using this board is to show the feasibility of proposed Deep SNNs. We want to learn; such SNN architectures could be implemented with similar ANN structures or not. We could not calculate the total power consumption of SNNs on the NVIDIA board. Because less accuracy and more processing time than ANNs leads to more power consumption for SNNs [32] on this board. To check the actual energy of the SNNs, we require neuromorphic chips such as TrueNorth, SpiNNaker, and Intel Loihi. In the literature, the authors of [32] mentioned that SNN consumes less power than ANNs. Neuromorphic architectures, due to non-von Neumann architectures, are more suitable for SNNs. Neuromorphic hardware has been designed to check the energy consumption of SNNs, and we will consider deploying all the proposed SNNs on such hardware in the future.

The above metrics are to be explored in conjunction with the customized SNN and ANN models on both PC and embedded systems to investigate the model training performance against existing literature. However, most works focused on PC-based performance evaluations, which must be validated against proposed model performances. However, due to the lack of literary works that employ deeper SNN directly trained using backpropagation and tested on embedded platforms, the results attained in the proposed work could serve as a benchmark for such research interests. To check the energy consumption of SNNs, we will use neuromorphic chips for better understandings and calculations in the future.

## 6. Discussion

In this work, we presented the possibility of directly training the deep convolutional spiking neural networks (DCSNNs) with surrogate gradient descent by following the deep neural network architectures such as VGG and ResNet. Furthermore, we emphasize that SNNs can be trained directly by considering trivial yet effective techniques compared to traditional ANNs. However, we also encounter the overfitting problem during training the SNNs. We show that by using the dropout technique, overfitting issues are to be resolve by applying small dropout ratios as compared to ANNs. We also observe that, during SNN training, by increasing the number of model layers, classification performance decline for the CIFAR-10 dataset. We then solve this issue by using the smaller number of output feature maps in convolutional layers for SNN based VGG model compared to the ANN. Moreover, we use fewer time-steps with surrogate gradient descent for deeper spiking neural networks, which leads to an increase in inference accuracy. In the case of CIFAR-10, we utilize 20 time-steps with SNN based VGG-13 network then the inference accuracy decline. We then change the number of time-steps from 20 to 15 with surrogate gradient descent to improve the classification performance.

A limitation of our work is that, with the growing number of layers in the SNN, classification results diminish. This avoids using the deeper models for inference, which will be the next step in this research direction. By conducting experiments, we reduce the loss with surrogate gradient descent. Still, we assume that the difference between this loss and the loss we want to reduce increases with the growing number of layers in the SNNs. In the future, this problem is considered for a deeper exploration. Additionally, training SNNs directly require somewhat different architectures rather than using the same architectures as ANNs. In this research work, we have implemented customized models similar to deep artificial neural structure, yet, more focused research has to be carried out to improve the performance of SNNs in all aspects.

## 7. Conclusions

To conclude this work, we proposed various deep convolutional SNNs trained with a surrogate gradient descent. These proposed SNN models achieved the best classification results on private as well as on public datasets. In the case of the four-layer SNN, we noticed that this network attains higher results than ANNs. We also used fewer inference time-steps with surrogate gradient descent, which leads us to efficient training and inference results in SNNs. Moreover, we resolved the overfitting problems by adding a small dropout ratio compared to traditional ANNs. We then deployed all the proposed models on an embedded platform to calculate the processing time and inference accuracy between the PC and the NVIDIA JETSON TX2 board.

As SNN accuracy still lags behind ANNs, in the future, we will design different architectures to implement SNNs with some customized backpropagation methods, which will reduce the training and inference processing time, as well as give a higher accuracy than ANNs. Moreover, we will deploy all the SNNs on neuromorphic chips such as TrueNorth, SpiNNaker and Intel Loihi to prove that SNNs consume less energy and processing time than ANNs.

## Figures and Tables

**Table 1:** Variables used in this research work.

Variables	Values
Training time-steps	10, 20, 30
Inference time-steps	Same as training time-steps
Membrane Potential time constant	10
Average pooling and stride ratio	2 × 2 and 2
Batch-size	8, 16 and 32
Neuron Threshold	1 and 0.5
Learning rate	0.0025 to 0.0033
Dropout value	0.2 to 0.25
Constant value for initialization of weights	1, 0

**Table 2:** Details of License Plate, KITTI, MNIST, SVHN and CIFAR-10 Dataset.

Datasets	Image Size	Training Examples	Testing Examples	Classes
Korean License Plate	32 × 32	40,000	10,000	50
KITTI	32 × 32	14,885	3722	6
MNIST	28 × 28	60,000	10,000	10
CIFAR-10	32 × 32	50,000	10,000	10
SVHN	32 × 32	73,000	26,000	10

**Table 3:** Proposed DCSNN models for MNIST, KITTI and License plate dataset.

Four-Layer Model			
**Model Layers**	**Filter Size**	**Number of Output Feature Maps**	**Stride**
Convolutional layer	1 × 3 × 3	32	1
Average Pooling Layer	2 × 2		2
Convolutional Layer	32 × 3 × 3	64	1
Average Pooling Layer	2 × 2		2
Fully Connected Layer		200	
Output Layer		10	
**VGG-6 Model**			
**Model Layers**	**Filter Size**	**Number of Output Feature Maps**	**Stride**
Convolutional Layer	1 × 3 × 3	32	1
Convolutional Layer	32 × 3 × 3	32	1
Average Pooling Layer	2 × 2		2
Convolutional Layer	32 × 3 × 3	64	1
Convolutional Layer	64 × 3 × 3	64	1
Average Pooling Layer	2 × 2		2
Fully Connected Layer		4096	
Output Layer		6	
**ResNet-6 Model**			
**Model Layers**	**Filter Size**	**Number of Output Feature Maps**	**Stride**
Convolutional layer	1 × 3 × 3		1
Average Pooling Layer	2 × 2	32	2
Convolutional Layer	32 × 3 × 3	64	1
Convolutional layer	64 × 3 × 3	64	1
Skip Connection	32 × 3 × 3	64	2
Convolutional Layer	64 × 3 × 3	128	1
Convolutional Layer	128 × 3 × 3	128	2
Skip Connection	64 × 3 × 3	128	2
Fully Connected Output Layer		50	

**Table 4:** Proposed DCSNN models for Street View House Number(SVHN) and CIFAR-10 dataset.

VGG-8 Model			
**Model layers**	**Filter size**	**Number of output feature maps**	**Stride**
Convolutional layer	32 × 3 × 3	64	1
Convolutional layer	64 × 3 × 3	64	2
Average pooling layer	2 × 2		
Convolutional layer	64 × 3 × 3	128	1
Convolutional layer	128 × 3 × 3	128	1
Average pooling layer	2 × 2		2
Convolutional layer	128 × 3 × 3	256	1
Convolutional layer	256 × 3 × 3	256	1
Average pooling layer	2 × 2		2
Fully connected layer		1024	
Output layer		10	
**VGG-11 Model**			
**Model layers**	**Filter size**	**Number of output feature maps**	**Stride**
Convolutional layer	3 × 3 × 3	64	1
Convolutional layer	64 × 3 × 3	64	1
Average pooling layer	2 × 2		2
Convolutional layer	64 × 3 × 3	128	1
Convolutional layer	128 × 3 × 3	128	1
Average pooling layer	2 × 2		2
Convolutional layer	128 × 3 × 3	256	1
Convolutional layer	256 × 3 × 3	256	1
Average pooling layer	2 × 2		2
Convolutional layer	256 × 3 × 3	256	1
Convolutional layer	256 × 3 × 3	512	1
Average pooling layer	2 × 2		2
Fully connected layer		1024	
Fully connected layer		1024	
Output layer		10	
**VGG-13 Model**			
**Model layers**	**Filter Size**	**Number of Output Feature Maps**	**Stride**
Convolutional Layer	3 × 3 × 3	64	1
Convolutional Layer	64 × 3 × 3	64	2
Average Pooling Layer	2 × 2		
Convolutional Layer	64 × 3 × 3	64	1
Convolutional Layer	128 × 3 × 3	128	1
Average pooling Layer	2 × 2		2
Convolutional Layer	128 × 3 × 3	128	1
Convolutional Layer	256 × 3 × 3	256	2
Average Pooling Layer	2 × 2		2
Convolutional Layer	256 × 3 × 3	256	1
Convolutional Layer	256 × 3 × 3	256	1
Average pooling Layer	2 × 2		2
Convolutional Layer	256 × 3 × 3	512	1
Convolutional Layer	512 × 3 × 3	512	1
Average Pooling Layer	2 × 2		2
Fully Connected Layer		1024	
Fully Connected Layer		1024	
Output Layer		10	

**Table 5:** SNN Classification Performance evaluation on MNIST and CIFAR-10 datasets.

Model	Learning Techniques	Accuracy MNIST	Accuracy CIFAR-10
[66]	Offline Learning, conversion	99.10%	-
[24]	Offline Learning, conversion	99.44%	88.82%
[67]	ANN2SNN	-	93.63%
[23]	Offline Learning, conversion	-	91.55%
[27]	Layer-wise STDP	98.40%	-
[32]	Spike-based BP	99.36%	-
[58]	Spike-based BP	99.46%	-
[39]	Spike-based BP	99.59%	90.55%
[57]	Spike-based BP	99.31%	-
[33]	Spike-based BP	99.42%	50.70%
[37]	Backpropagation	99.40%	90.20%
**This Work**	**Surrogate Gradient Descent**	**99.66%**	**91.58%**

**Table 6:** Comparison of optimized parameters of our work with SOTA.

Method	MNIST Accuracy	CIFAR-10 Accuracy	Threshold Value	Optimizer	Time Steps	Learning Rate	Batch Size
[58]	99.46%	-	1	ADAM	300	0.0001	64
[33]	99.42%	50.70%	1.5	SGD, ADAM	30	0.5	100
[23]	-	91.55%	1.5	SGD	2500	0.005	256
[37]	99.40%	90.20%	1	ADAMW	10, 20, 40	0.0005	32, 64
[39]	99.59%	90.55%	1	SGD	50, 100	0.002, 0.003	16, 32
**Ours**	**99.50%**	**91.58%**	**1 and 0.5**	**ADAM, SGD**	**8, 10, 15**	**0.00285, 0.0033**	**16, 32, 64**

**Table 7:** Processing time and inference accuracy of different datasets on PC and NVIDIA JETSON TX2.

		SNN Performance				ANN Performance			
**Dataset**	**Proposed Model**	**Accuracy**		**Processing Time (per Image)**		**Accuracy**		**Processing Time (per Image)**	
		PC	NVIDIA TX2	PC	NVIDIA TX2	PC	NVIDIA TX2	PC	NVIDIA TX2
MNIST	4-layer SNN	99.66%	99.66%	0.16 ms	1.93 ms	99.31%	99.31%	0.06 ms	0.07 ms
KITTI	VGG-6	95.03%	99.27%	3.12 ms	13.2 ms	98.01%	98.01%	0.10 ms	0.010 ms
License Plate	ResNet-6	96.46%	95.01%	6.0 ms	21.0 ms	93.57%	94.01%	0.75 ms	2.08 ms
	ResNet-7	93.68%	93.84%	6.3 ms	21.5 ms	94.37%	94.40%	0.80 ms	2.07 ms
SVHN	VGG-8	94.01%	95%	4.3 ms	13.5 ms	93.70%	93.70%	0.30 ms	1.35 ms
	VGG-9	93.68%	93.68%	4.5 ms	13.60 ms	92.08%	92.08%	0.30 ms	1.40 ms
CIFAR-10	VGG-11	91.25%	91.25%	8.2 ms	23.3 ms	91.03%	91.55%	0.070 ms	0.15 ms
	VGG-13	91.58%	91.43%	11.3 ms	25.2 ms	92.08%	92.50%	0.80 ms	0.45 ms

## Data Availability

Not applicable.
